# Evaluation of Factors Associated with Adverse Drug Events in South Korea Using a Population-Based Database

**DOI:** 10.3390/jcm11216248

**Published:** 2022-10-23

**Authors:** Eunkyeong Choi, Siin Kim, Hae Sun Suh

**Affiliations:** 1 College of Pharmacy, Pusan National University, Busan 46241, Korea; 2 College of Pharmacy, Kyung Hee University, Seoul 02447, Korea; 3 Department of Regulatory Science, Graduate School, Kyung Hee University, Seoul 02447, Korea

**Keywords:** adverse drug events, risk factor, claims data, multivariate logistic regression

## Abstract

This retrospective study aims to investigate the factors associated with the occurrence of ADEs using nationally representative claims data. All patients with at least one claim with diagnosis codes denoting potential ADE between 1 July 2015 and 31 December 2015 were included. Potential ADE was defined as ADE identified in the claims data, because it was not verified. The index date was defined as the date of the first claim with potential ADEs. Demographic data were collected at the index date, while data on comorbidities and number of medications used were collected six months before the index date. Multivariate logistic regression was used to explore the association between potential ADEs and several factors, including sex, age group, insurance type, comorbidities, and number of prescribed medications. Patients with potential ADEs were older, had more chronic diseases, and used more medications than those without potential ADEs. In the multivariate analysis, occurrence of potential ADEs was associated with age (≥65 years, odds ratio [OR] 1.15, 95% confidence interval [CI] 1.08–1.21), Medical Aid program (OR 1.37, 95% CI 1.27–1.47), Charlson Comorbidity Index scores (≥5, OR 2.87, 95% CI 2.56–3.20), and use of six or more medications (6–10 medications, OR 1.89, 95% CI 1.79–1.99). Age, Medical Aid program, comorbidities, and number of medications were associated with occurrence of potential ADEs.

## 1. Introduction

With increasing drug use, adverse drug events (ADEs) have become a critical public health concern. In previous studies, approximately 5–10% of patients had ADEs, which increased the risk of death, and the costs associated with ADEs were substantial [1,2,3,4,5,6,7,8]. Furthermore, nearly one third of ADEs are considered preventable and are more likely to be serious and life-threatening events [5]. A study conducted in the late 1990s in two tertiary hospitals in the USA reported that annual costs attributable to preventable ADEs were approximately $2.8 million [5,6,7]. In addition, a recent systematic review of 18 observational studies conducted in the USA and Europe found that the estimated costs of preventable ADEs reached €9000 [9].

One potentially effective strategy for preventing ADEs is identifying those patients at risk of ADEs. Despite concerns that ADEs contribute to significant clinical and economic problems, little is known about the factors associated with ADEs among Asian populations, including the South Korean population. According to a number of studies conducted in Western countries, important independent risk factors for ADEs include sex, age, comorbidities, polypharmacy, health service utilization, and inappropriate drug use [10,11,12]. However, risk factors for ADE may differ according to the characteristics of the study population, including culture, economic status, and patterns of drug use. For instance, a study conducted in 81 hospitals in Italy reported that female gender was a risk factor for ADR-related hospital admissions [13]. However, a prospective observational study conducted in two hospitals in Spain found that sex was not associated with ADEs [14]. Moreover, previous studies determined that risk factors related to the occurrence of ADEs were restricted to hospitals representing a local population or specific age groups [11,15,16]. Previous studies in South Korea that explored risk factors associated with ADEs were often limited to genetic factors for specific drugs [17,18]. Thus, these results are difficult to generalize in clinical practice. The proportion of older adults in South Korea is increasing rapidly, and, similarly to other countries in Asia, South Korea has a cultural preference for consuming health supplements such as herbal medicines [19,20]. Therefore, the risk factors associated with ADEs in South Korea may differ from those reported in previous studies conducted in Western countries. Understanding the factors associated with ADEs will aid in prioritizing policy activities for ADE prevention.

Therefore, this retrospective study was conducted using population-based data to investigate the associated factors for ADEs in South Korea.

## 2. Materials and Methods

### 2.1. Data Source

We used the National Patient Sample database from the Health Insurance Review and Assessment Service (HIRA-NPS) from 1 January 2015 to 31 December 2015. The HIRA-NPS database is a secondary data source of sex- and age-stratified random samples (approximately 1,400,000 persons) from health insurance claims data covering approximately 50 million South Korean enrollees. Based on a validation study, the HIRA-NPS database provided the national representativeness of all enrollees [21,22]. In South Korea, there are two government-run mandatory national health security systems: The National Health Insurance (NHI) program is a wage-based, contributory insurance program that covers 96% of the population; the Medical Aid (MA) program is a government-subsidized public health assistance program for low-income households and individuals who are unable to pay for health care [23]. The HIRA-NPS database provides claims records for all types of healthcare services, including outpatient care, inpatient care, emergency department visits, diagnoses, prescribed medications, and sociodemographic information of enrollees, including sex, age, and type of health insurance [21]. The International Classification of Diseases, Tenth Revision (ICD-10) was recorded as diagnoses in the database. Individuals could not be identified because data are anonymized [24].

### 2.2. Study Subjects

**Figure 1:**
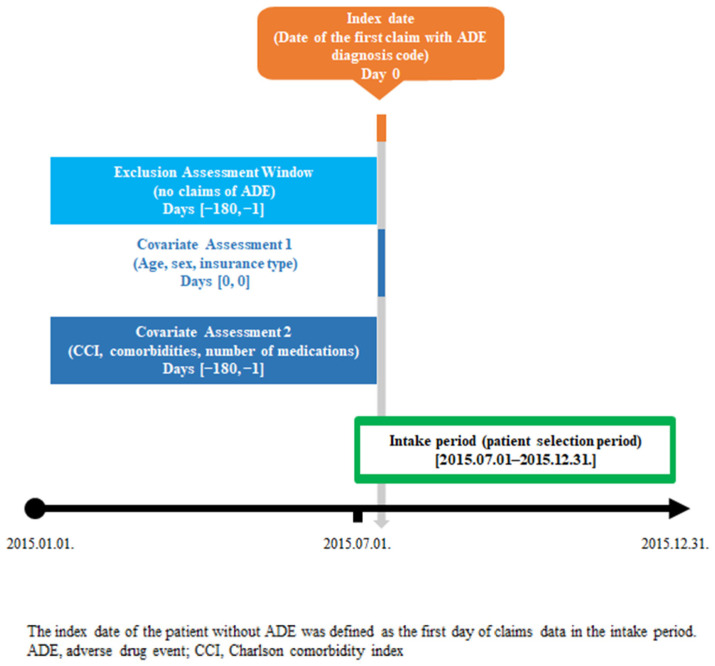
Study scheme.

Patients were identified as having a potential ADE if they had at least one claim record of ADE diagnosis codes during a six-month intake period from 1 July to 31 December 2015. Figure 1 shows the study scheme used in this study. We defined the index date for patients with potential ADE as the date of the first ADE claim during the intake period. For patients without potential ADE, we defined the index date as the date of the first claim record during the intake period. We also obtained baseline characteristics of the study population, such as comorbidities and number of prescribed medications, six months from the pre-index period before the index date. To identify patients with new cases of potential ADE, individuals were excluded if they had at least one claim record of ADE within the six months before the index date.

### 2.3. Identification of Potential ADEs

In the selection of patients with ADE, we defined an ADE as “harm caused by a drug or the inappropriate use of a drug” [25]. Compared to adverse drug reactions, defined as “harmful and unintended consequences occurring due to appropriate use of a drug,” ADE consists of a broad spectrum of events or reactions [26]. Thus, we included adverse effects of therapeutic use, poisoning, failure of medical care, and medication errors.

To identify ADEs in the claims data, we used ICD-10 codes that met one of the following criteria based on the previous study: (1) code description includes the phrase “caused by a drug” or “caused by a drug or other substance”; (2) code description includes the phrase “poisoning by a drug” or “poisoning by a drug or other substance”; (3) code description includes the phrase “caused by vaccine”; and (4) code description does not refer to a drug but implies as “ADE very likely” [27]. Finally, we included 586 ICD-10 codes that identified ADEs (Supplementary Table S1). However, we did not verify whether identified ADEs in the claims data were true ADEs in the patients’ original health records. Therefore, we defined it as potential ADEs.

### 2.4. Statistical Analysis

To compare the baseline characteristics of patients with and without potential ADE, we used the Student *t*-test for continuous variables and chi-square test or Fisher’s exact test for categorical variables. A *p*-value < 0.05 indicated a difference in the characteristics between the two groups.

To identify the factors associated with the occurrence of potential ADEs, we performed univariate logistic analysis to explore the potentially important variables to be used in the subsequent multivariate logistic regression. We included variables and categorized them as follows: sex (male or female), age group (<20, 20–44, 45–64, and ≥65 years), insurance type (NHI or MA), Charlson Comorbidity Index score (0, 1, 2, 3, 4, and ≥5), comorbidities, and number of medications (<6, 6–10, 11–20, and ≥21). We obtained odds ratios (ORs) and 95% confidence intervals (CIs) after adjusting for sex, age group, insurance type, Charlson Comorbidity Index score, comorbidities, and number of prescribed medications within six months from the index date. The Charlson Comorbidity Index score is a standard measure of disease burden, and a high index score indicates poor health conditions [28]. According to Quan’s coding algorithms, we calculated the Charlson Comorbidity Index score by analyzing all diagnosis codes within six months from the index date [29]. Comorbidities included in the Charlson Comorbidity Index were measured within six months from the index date. To measure the number of prescribed medications within six months, we collected the Korean national drug codes denoting the general name code of drugs and counted the number of unique active drug ingredients per patient within six months, whether different drugs were prescribed on the same day or on different days. The code consists of nine digits, including six numbers and three English letters. The active ingredient of a medication can be identified by the first four digits of the Korean national drug codes. Accordingly, we defined different medications by identifying distinct active ingredients. All variables used in the present study were identified in previous studies as factors influencing ADEs [12,30,31].

All analyses were performed using the SAS statistical software (version 9.4; SAS Institute Inc., Cary, NC, USA).

## 3. Results

During the intake period, from 1 July 2015 to 31 December 2015, the study population was 1,326,638. Of these, 15,713 (1.18%) individuals were identified as new patients with potential ADEs who met the eligibility criteria. The numbers of patients with potential ADEs who had outpatient, inpatient, and emergency department visits were 12,612 (80.26%), 2093 (13.32%), and 1008 (6.42%), respectively. There were significant differences in most baseline characteristics among patients with and without potential ADEs (Table 1).

Compared to patients without potential ADEs, patients with potential ADEs were older and more frequently enrolled in the MA program. Furthermore, the average Charlson Comorbidity Index scores and number of prescribed medications within 6 months in patients with potential ADEs were significantly higher than in those without potential ADEs.

To investigate independent associations with potential ADEs, we conducted a multivariate logistic regression analysis followed by univariate logistic regression analysis (Table 2). We found that independent factors associated with potential ADEs were age ≥ 20 years (20–44 years, OR 1.24, 95% CI 1.18–1.30; 45–64 years, OR 1.17, 95% CI 1.12–1.23; ≥65 years, OR 1.09, 95% CI 1.03–1.16) and MA enrollees (OR 1.35, 95% CI 1.25–1.45). Female sex showed a relationship with potential ADEs in the univariate analysis, but this correlation was not observed after controlling for age, insurance type, Charlson Comorbidity Index score, and number of prescribed medications.

Particularly, comorbidities and number of medications had considerable association with the occurrence of potential ADEs. Moreover, there were increasing patterns of ORs for potential ADEs with higher Charlson Comorbidity Index scores and number of medications. Chronic diseases significantly associated with the occurrence of potential ADEs were connective tissue/rheumatic disease (OR 1.22, 95% CI 1.11–1.34), peptic ulcer disease (OR 1.08, 95% CI 1.02–1.13), mild liver disease (OR 1.30, 95% CI 1.24–1.37), moderate-to-severe liver disease (OR 1.56, 95% CI 1.18–2.06), diabetes without complications (OR 1.12, 95% CI 1.06–1.17), cancer (OR 1.15, 95% CI 1.05–1.25), and metastatic carcinoma (OR 1.43, 95% CI 1.17–1.76).

## 4. Discussion

This retrospective study investigated the association of patient characteristics, disease, and medication with the occurrence of potential ADEs using nationally representative claims data. Multivariate logistic regression analysis revealed that age, MA program, comorbidities, and number of medications were associated with potential ADEs after controlling for baseline characteristics of the study population.

We defined the study outcomes as ADEs to comprehensively capture the harm related to drug use. Adverse drug reaction (ADR), often used as another research outcome of adverse effects associated with medication, involves harmful and unintended consequences that occur at doses normally used in humans for prophylaxis, diagnosis, or treatment of disease [26]. However, ADR does not include clinically significant issues, such as poisoning and medication errors, because it involves appropriate drug use only. For example, hearing loss due to overdose of a potential drug would not be considered an ADR but an ADE. Moreover, the term ADE is preferable because the diagnosis code cannot differentiate the cause of adverse effects in the claims data [32].

Our results showed that sex was not associated with potential adverse effects. This finding is consistent with previous results [11,33]. However, female gender was frequently reported as an independent factor for ADEs [13,16,34,35,36]. A prospective multicenter study in Europe reported that women had a higher risk of ADE occurrence than men, as reflected in the significant OR of 1.60 (95% CI 1.31–1.94) [36]. In a study conducted in the UK, including individuals aged ≥65 years, female gender was associated with a higher occurrence of ADEs [16]. Potential reasons for ADE risk in women are differences in physical (body fat and organ function) and physiological features (pregnancy and menopause), as well as differences in pharmacodynamics (effects of drugs) and pharmacokinetics (absorption, distribution, metabolism, and excretion) [37,38,39,40]. However, other studies reported that male gender was an independent factor for ADEs [15,41].

Several studies have already found an apparent increase in ADE risk with age [11,16,33,42,43]. A possible explanation for the effect of age on the occurrence of ADE is that the physiological changes associated with advancing age may render individuals vulnerable to adverse impacts [44]. However, there have been inconsistent findings regarding the effect of age on the occurrence of ADEs [11,45,46]. For instance, a multicenter survey that included internal medicine and geriatric wards reported that age was not a significant predictor of ADEs [45]. Conversely, a study conducted in two Dutch hospitals reported that patients aged ≥80 years had lower risk of occurrence of ADEs than those aged ≤60 years [46]. Interestingly, in our study, the risk of potential ADEs showed a tendency to decrease with increasing age after adjusting for the characteristics of the study patients. Further studies are required to explore the causes of the different effects of age on the occurrence of ADEs.

Patients enrolled in the MA program showed a higher OR for the occurrence of potential ADEs than those enrolled in the NHI program. A 2014 study in South Korea reported that polypharmacy was associated with MA enrollees after controlling for sex, age, and chronic diseases [47]. They also expected to use excessive healthcare utilization because of having a low perception of efficient use of healthcare resources [48]. Although we did not consider healthcare utilization, such as history of hospitalization, outpatient visits, and emergency department visits in this study, service and drug utilization patterns might be a potential reason for the effects on the occurrence of ADEs.

The important finding of this study is that comorbidities and number of medications showed a significant association with the occurrence of potential ADEs. Patients with chronic comorbidities have an increased likelihood of polypharmacy [49]. Several previous studies frequently reported that polypharmacy was an important independent predictor for ADEs [11,13,16]. For example, according to a study in New England, USA, Medicare enrollees using eight or more medications showed a 2.9 odds of ADEs compared to those with one or fewer medications [16]. Furthermore, concomitant drug use was considerably associated with an increased risk of serious adverse effects [50]. A potential reason for the impact of polypharmacy on ADEs might be that using several medications may increase the probability of drug–drug interactions and inappropriate drug use [51]. Furthermore, in this study, patients with moderate-to-severe liver disease and metastatic cancer were highly associated with the occurrence of potential ADEs compared to other chronic conditions. Impaired liver function may influence the pharmacokinetics and pharmacodynamics of drugs, as most drugs are predominantly metabolized in the liver. For instance, lower hepatic blood flow may affect drug clearance, and hypoalbuminemia can cause increased concentrations of highly protein-bound drugs. Patients with metastatic cancer receive chemotherapy to delay disease progression and improve their quality of life. However, cancer chemotherapy also causes various severe adverse effects, such as neutropenia, vomiting, diarrhea, and weakness. Furthermore, these patients are frequently exposed to drug–drug interactions because they receive a high number of medications and are significantly susceptible to adverse effects due to their intensive medical treatment [52,53,54]. Therefore, physicians and pharmacists should be more aware of potential drug–drug interactions and pay attention to ADEs in fragile patients.

A better understanding of individual factors associated with ADE is crucial for improving patient safety. In the present study, older adults, MA program enrollees, and patients with polypharmacy or high comorbid conditions were at greater risk of potential ADEs. Our findings highlight the importance of health policy activities in prioritizing ADE prevention for vulnerable patients.

To the best of our knowledge, this is the first study to explore factors associated with the occurrence of potential ADEs using a representative database that appropriately reflects the entire South Korean population. The HIRA-NPS data contain valuable healthcare resource information, including sociodemographic characteristics, diagnoses, and drug utilization. Therefore, claims data have the advantage of comprehensively investigating the association between individual-level factors, such as disease and medication, and occurrence of ADEs. The International Classification of Diseases, eleventh Revision (ICD-11) was adopted in 2019 and implemented worldwide. Therefore, to identify ADEs using the ICD-11 codes, it is necessary to perform direct mapping from ICD-10 to ICD-11 and determine whether the individually matched ICD-11 codes indicate an ADE. Furthermore, the ICD-11 codes provide more health information; therefore, we expect that more ADEs can be detected using the ICD-11 codes in the claims data. We suggest that further studies should be conducted on the epidemiology of ADEs using the ICD-11 codes.

However, this study has several limitations. Firstly, as with other previous studies using administrative databases, we could not capture all ADEs, including abnormal laboratory values, because limited clinical information was available in the claims data. Furthermore, there is a limitation in detecting patients with ADEs owing to the restricted diagnosis codes indicating ADEs. Several approaches to identify ADE have been developed, including spontaneous reporting, chart review, computerized monitoring systems, and administrative data [55]. Spontaneous reporting is the most widely used method, but under-reporting is a problem that limits its effectiveness. Chart review is the gold standard, but it is time- and cost-intensive. Computerized monitoring systems, compared to chart reviews, are more efficient in identifying ADEs, but it is challenging to develop algorithms with high-specificity signals. Administrative data have emerged as an alternative for the identification of ADEs. Compared to other methods, it is relatively inexpensive and readily available. However, no specific methodology has been applied for ADE detection. Based on previous studies, we believe that identifying ADEs using diagnosis codes associated with ADE can be an alternative method for pharmacovigilance [56,57,58]. Administrative data can be used to identify ADEs at the population level [57]. The second limitation is that we were not able to identify whether individuals were using over-the-counter drugs, dietary supplements, or herbal medicines that were not covered by health coverage. Moreover, with our definition of the number of medications, we included both drugs used regularly for chronic disease and those used in the short-term for acute disease. Hence, the effects of the number of medications on the occurrence of ADEs may differ from actual estimates. Thirdly, as the claims data were collected for accounting purposes, the effect of coding quality on identifying ADEs is uncertain. Therefore, some ADEs in the claims data may not have been true ADEs, and the majority of mild ADEs may have been missed because they were incompletely documented. Therefore, careful interpretation should be required to understand the risk factors of ADEs in our results. In addition, the reliability and validity of the claims data may limit the generalization of our results. As suggested by previous studies, validation of codes related to ADEs is necessary to enhance sensitivity and specificity of ADEs detection using ICD codes [27,59]. In addition, validation needs to be performed in various healthcare settings (outpatient, inpatient, and emergency department) on the different severities of ADEs (mild, moderate, and severe) to understand the risk factors of ADEs in the general population. Fourthly, the data source used in our study was not recent. Therefore, the factors associated with ADE may differ from the current estimates. However, several diagnosis codes that are classified as sensitive information are not provided in the HIRA-NPS databases established after 2015. Therefore, we used the 2015 database, which is the most recent database in which diagnosis codes could be fully identified. Lastly, other potential factors, such as lifestyle (alcohol consumption, smoking status), compliance with therapy, healthcare service utilization, and genetic features, were not considered in the present study. Therefore, unmeasured cofounders may have been present in our results. Nevertheless, we included frequently reported factors such as patient-, disease-, and medication-related characteristics [12], and most of our findings were consistent with those of previous studies.

## 5. Conclusions

This study evaluates the sociodemographics, comorbidities, and number of medications associated with potential ADEs in South Korea using population-based data. Our study demonstrates that increased age, MA enrollment, high number of comorbidities, and high number of medications used are all independent factors of occurrence of potential ADEs. This finding indicates that, to prevent and reduce ADEs, healthcare professionals and policymakers should keep in mind those who have a higher risk of developing ADEs, particularly those with a high disease burden.

## Figures and Tables

**Table 1:** Baseline characteristics of the study population.

	Patients with Potential ADE (*n* = 15,713)		Patients without Potential ADE (*n* = 1,289,319)		*p*-Value ^a^
Age, mean (SD)	44.70	(22.76)	40.23	(21.69)	<0.01
Sex, No. (%)					
Male	7016	(44.65)	615,037	(47.70)	<0.01
Female	8697	(55.35)	674,282	(52.30)	
Insurance type, No. (%)					
NHI program	14,865	(94.60)	1,252,005	(97.11)	<0.01
MA program	848	(5.40)	37,314	(2.89)	
Charlson Comorbidity Index score, mean (SD)	0.81	(1.45)	0.39	(1.22)	<0.01
Comorbidities, No. (%)					
Myocardial infarction	138	(0.88)	5415	(0.42)	<0.01
Congestive heart failure	487	(3.10)	18,846	(1.46)	<0.01
Peripheral vascular disease	1132	(7.20)	51,523	(4.00)	<0.01
Cerebrovascular disease	935	(5.95)	41,498	(3.22)	<0.01
Dementia	485	(3.09)	19,668	(1.53)	<0.01
Chronic pulmonary disease	3390	(21.57)	179,227	(13.90)	<0.01
Connective tissue/rheumatic disease	486	(3.09)	16,548	(1.28)	<0.01
Peptic ulcer disease	2316	(14.74)	95,949	(7.44)	<0.01
Liver disease, mild	2306	(14.68)	91,797	(7.12)	<0.01
Liver disease, moderate to severe	54	(0.34)	1337	(0.10)	<0.01
Diabetes without complications	2114	(13.45)	96,600	(7.49)	<0.01
Diabetes with complications	767	(4.88)	32,647	(2.53)	<0.01
Hemiplegia or paraplegia	106	(0.67)	3931	(0.30)	<0.01
Renal disease	228	(1.45)	7860	(0.61)	<0.01
Cancer	959	(6.10)	31,478	(2.44)	<0.01
Metastatic carcinoma	183	(1.16)	2727	(0.21)	<0.01
HIV/AIDS	4	(0.03)	53	(0.00)	<0.01
Number of medications, mean (SD)	17.82	(14.98)	10.33	(11.08)	<0.01

SD, standard deviation; NHI, National Health Insurance; MA, Medical Aid; HIV, human immunodeficiency virus; AIDS, acquired immunodeficiency syndrome ^a^ Student *t*-test for continuous variables and chi-square test or Fisher’s exact test for categorical variables were used.

**Table 2:** Unadjusted and adjusted odds ratio of potential adverse drug events for characteristics of the study population.

Characteristics	Unadjusted Odds Ratio (95% CI)	*p*-Value	Adjusted Odds Ratio (95% CI) ^a^	*p*-Value
Sex				
Male (reference)	-	<0.01	-	0.48
Female	1.13 (1.10–1.17)		1.01 (0.98–1.04)	
Age group, years				
<20 (reference)	-	<0.01	-	<0.01
20–44	1.07 (1.02–1.12)		1.25 (1.19–1.32)	
45–64	1.36 (1.30–1.43)		1.21 (1.16–1.27)	
≥65	1.87 (1.78–1.97)		1.15 (1.08–1.21)	
Insurance type				
NHI program (reference)	-	<0.01	-	<0.01
MA program	1.92 (1.79–2.05)		1.37 (1.27–1.47)	
Charlson Comorbidity Index score				
0 (reference)	-	<0.01	-	<0.01
1	1.80 (1.73–1.87)		1.09 (1.05–1.14)	
2	2.25 (2.13–2.39)		1.22 (1.14–1.30)	
3	3.09 (2.85–3.34)		1.56 (1.43–1.70)	
4	3.62 (3.21–4.08)		1.73 (1.53–1.96)	
≥5	6.13 (5.53–6.80)		2.87 (2.56–3.20)	
Comorbidities ^b^				
Myocardial infarction	2.10 (1.78–2.49)	<0.01	0.98 (0.83–1.17)	0.84
Congestive heart failure	2.16 (1.97–2.37)	<0.01	1.02 (0.93–1.12)	0.71
Peripheral vascular disease	1.87 (1.76–1.98)	<0.01	1.01 (0.94–1.07)	0.85
Cerebrovascular disease	1.90 (1.78–2.03)	<0.01	0.90 (0.83–0.97)	<0.01
Dementia	2.06 (1.88–2.26)	<0.01	1.04 (0.94–1.15)	0.41
Chronic pulmonary disease	1.70 (1.64–1.77)	<0.01	0.80 (0.76–0.84)	<0.01
Connective tissue/rheumatic disease	2.46 (2.24–2.69)	<0.01	1.22 (1.11–1.34)	<0.01
Peptic ulcer disease	2.15 (2.06–2.25)	<0.01	1.08 (1.02–1.13)	<0.01
Liver disease, mild	2.24 (2.15–2.35)	<0.01	1.30 (1.24–1.37)	<0.01
Liver disease, moderate to severe	3.32 (2.53–4.36)	<0.01	1.56 (1.18–2.06)	<0.01
Diabetes without complications	1.92 (1.83–2.01)	<0.01	1.12 (1.06–1.17)	<0.01
Diabetes with complications	1.98 (1.84–2.13)	<0.01	1.07 (0.99–1.15)	0.10
Hemiplegia or paraplegia	2.23 (1.83–2.70)	<0.01	0.86 (0.71–1.06)	0.16
Renal disease	2.40 (2.10–2.74)	<0.01	0.91 (0.79–1.04)	0.17
Cancer	2.60 (2.43–2.78)	<0.01	1.15 (1.05–1.25)	<0.01
Metastatic carcinoma	5.56 (4.78–6.46)	<0.01	1.43 (1.17–1.76)	<0.01
HIV/AIDS	6.20 (2.24–17.12)	<0.01	1.48 (0.53–4.14)	0.45
Number of medications				
<6 (reference)	-	<0.01	-	<0.01
6–10	1.92 (1.82–2.02)		1.89 (1.79–1.99)	
11–20	2.80 (2.67–2.93)		2.69 (2.56–2.82)	
≥21	4.72 (4.51–4.94)		4.05 (3.84–4.27)	

CI, confidence interval; NHI, National Health Insurance; MA, Medical Aid; HIV, human immunodeficiency virus; AIDS, acquired immunodeficiency syndrome. ^a^ Estimates from the logistic regression model included variables for sex, age group, insurance type, Charlson Comorbidity Index score, and number of medications. ^b^ For odds ratio, using patients with no history of comorbidity as the reference category.

## Data Availability

Not applicable.

## Supplementary Materials

The following supporting information can be downloaded at: https://www.mdpi.com/article/10.3390/jcm11216248/s1, Table S1: List of the International Classification of Diseases, Tenth Revision (ICD-10) codes associated with adverse drug events.
