# Supplementary material for: Evaluation of Factors Associated with Adverse Drug Events in South Korea Using a Population-Based Database
Source: J Clin Med. 2022 Oct 23;11(21):6248. doi: 10.3390/jcm11216248 (PMC9657773; doi:10.3390/jcm11216248)
Supplement: Supplementary file 1 [file jcm-11-06248-s001.zip › jcm-1929697-supplementary.pdf]

Table S1. List of the International Classification of Diseases, Tenth Revision (ICD-10) codes associated with adverse drug events.

ICD-10 code	Description
Drug-induced	
D52.1	Drug-induced folate deficiency anaemia
D59.0	Drug-induced autoimmune haemolytic anaemia
D59.2	Drug-induced nonautoimmune haemolytic anaemia
D61.1	Drug-induced aplastic anaemia
D64.2	Secondary sideroblastic anaemia due to drugs and toxins
D68.3	Hemorrhagic disorder due to circulating anticoagulants
E03.2	Hypothyroidism due to medicaments and other exogenous substances
E06.4	Drug-induced thyroiditis
E16.0	Drug-induced hypoglycaemia without coma
E23.1	Drug-induced hypopituitarism
E24.2	Drug-induced Cushing’s syndrome
E27.3	Drug-induced adrenocortical insufficiency
E66.1	Drug-induced obesity
F11*	Mental and behavioural disorders due to use of opioids
F13*	Mental and behavioural disorders due to use of sedatives or hypnotics
F15*	Mental and behavioural disorders due to use of other stimulants, including caffeine
F19*	Mental and behavioural disorders due to multiple drug use and use of other psychoactive substances
G21.0	Malignant neuroleptic syndrome
G21.1	Other drug-induced secondary parkinsonism
G21.2	Secondary parkinsonism due to other external agents
G24.0	Drug-induced dystonia
G25.1	Drug-induced tremor
G25.4	Drug-induced chorea
G25.6	Drug-induced tics and other tics of organic origin
G44.4	Drug-induced headache, not elsewhere classified
G62.0	Drug-induced polyneuropathy
G72.0	Drug-induced myopathy
H26.3	Drug-induced cataract
H40.6	Glaucoma secondary to drugs
I42.7	Cardiomyopathy due to drugs and other external agents
I95.2	Hypotension due to drugs
J70.2	Acute drug-induced interstitial lung disorders
J70.3	Chronic drug-induced interstitial lung disorders
J70.4	Drug-induced interstitial lung disorders, unspecified
K85.3	Drug-induced pancreatitis
L10.5	Drug-induced pemphigus
L23.3	Allergic contact dermatitis due to drugs in contact with skin
L24.4	Irritant contact dermatitis due to drugs in contact with skin
L25.1	Unspecified contact dermatitis due to drugs in contact with skin
L27.0	Generalized skin eruption due to drugs and medicaments
L27.1	Localized skin eruption due to drugs and medicaments
L27.8	Dermatitis due to other substances taken internally
L27.9	Dermatitis due to unspecified substance taken internally
L43.2	Lichenoid drug reaction
L56.0	Drug phototoxic response
L56.1	Drug photoallergic response
L64.0	Drug-induced androgenic alopecia
M10.2	Drug-induced gout
M32.0	Drug-induced systemic lupus erythematosus
M34.2	Systemic sclerosis induced by drugs and chemicals
M80.4	Drug-induced osteoporosis with pathological fracture
M81.4	Drug-induced osteoporosis without pathological fracture
M83.5	Other drug-induced osteomalacia in adults
M87.1	Osteonecrosis due to drugs

N14.0	Analgesic nephropathy
N14.1	Nephropathy induced by other drugs, medicaments and biological substances
N14.2	Nephropathy induced by unspecified drug, medicament or biological substance
N14.4	Toxic nephropathy, not elsewhere classified
O35.5	Maternal care for (suspected) damage to fetus by drugs
O74.4	Toxic reaction to local anaesthesia during labour and delivery
P04.0	Fetus and newborn affected by maternal anaesthesia and analgesia in pregnancy, labour and delivery
P04.1	Fetus and newborn affected by other maternal medication
P04.4	Fetus and newborn affected by maternal use of drugs of addiction
P58.4	Neonatal jaundice due to drugs or toxins transmitted from mother or given to newborn
P93	Reactions and intoxications due to drugs administered to fetus and newborn
P96.1	Neonatal withdrawal symptoms from maternal use of drugs of addiction
P96.2	Withdrawal symptoms from therapeutic use of drugs in newborn
Q86.1	Fetal hydantoin syndrome
Q86.2	Dysmorphism due to warfarin
R50.2	Drug-induced fever
T78.2	Anaphylactic shock, unspecified
T78.3	Angioneurotic oedema
T78.4	Allergy, unspecified
T78.8	Other adverse effects, not elsewhere classified
T78.9	Adverse effect, unspecified
T80*	Complications following infusion, transfusion and therapeutic injection
T88.3	Malignant hyperthermia due to anaesthesia
T88.6	Anaphylactic shock due to adverse effect of correct drug or medicament properly administered
T88.7	Unspecified adverse event of drug or medicament
Y40*	Drugs, medicaments, and biological substances causing adverse effects in therapeutic use - systemic antibiotic
Y41*	Drugs, medicaments, and biological substances causing adverse effects in therapeutic use - other systemic anti-infectives and antiparasitics
Y42*	Drugs, medicaments, and biological substances causing adverse effects in therapeutic use - Hormones and their synthetic substitutes and antagonists, not elsewhere classified
Y43*	Drugs, medicaments and biological substances causing adverse effects in therapeutic use - Primarily systemic agents
Y44*	Drugs, medicaments and biological substances causing adverse effects in therapeutic use - Agents primarily affecting blood constituents
Y45*	Drugs, medicaments and biological substances causing adverse effects in therapeutic use - Analgesics, antipyretics and anti-inflammatory drugs
Y46*	Drugs, medicaments and biological substances causing adverse effects in therapeutic use - Antiepileptics and antiparkinsonism drugs
Y47*	Drugs, medicaments and biological substances causing adverse effects in therapeutic use - Sedatives, hypnotics and antianxiety drugs
Y48*	Drugs, medicaments and biological substances causing adverse effects in therapeutic use - Anaesthetics and therapeutic gases
Y49*	Drugs, medicaments and biological substances causing adverse effects in therapeutic use - Psychotropic drugs, not elsewhere classified
Y50*	Drugs, medicaments and biological substances causing adverse effects in therapeutic use - Central nervous system stimulants, not elsewhere classified
Y51*	Drugs, medicaments and biological substances causing adverse effects in therapeutic use - Drugs primarily affecting the autonomic nervous system
Y52*	Drugs, medicaments and biological substances causing adverse effects in therapeutic use - Agents primarily affecting the cardiovascular system
Y53*	Drugs, medicaments and biological substances causing adverse effects in therapeutic use - Agents primarily affecting the gastrointestinal system
Y54*	Drugs, medicaments and biological substances causing adverse effects in therapeutic use - Agents primarily affecting water-balance and mineral and uric acid metabolism

Y55*	Drugs, medicaments and biological substances causing adverse effects in therapeutic use - Agents primarily acting on smooth and skeletal muscles and the respiratory system
Y56*	Drugs, medicaments and biological substances causing adverse effects in therapeutic use - Topical agents primarily affecting skin and mucous membrane and ophthalmological, otorhinolaryngological and dental drugs
Y57*	Drugs, medicaments and biological substances causing adverse effects in therapeutic use -Other and unspecified drugs and medicaments
Y59*	Drugs, medicaments and biological substances causing adverse effects in therapeutic use - Other and unspecified vaccines and biological substances
Y88.0	Sequelae of adverse effects caused by drugs, medicaments and biological substances in therapeutic use
Z03.6	Observation for suspected toxic effect from ingested substance

Poisoning by drug

F55	Abuse of non-dependence-producing substances
T36*	Poisoning by systemic antibiotics
T37*	Poisoning by other systemic anti-infectives and anti-parasitics
T38*	Poisoning by hormones and their synthetic substitutes and antagonists, not elsewhere classified
T39*	Poisoning by nonopioid analgesics, antipyretics and antirheumatics
T40*	Poisoning by narcotics and psychodysleptics
T41*	Poisoning by anaesthetics and therpeutic gases
T42*	Poisoning by antiepileptic, sedative-hypnotic and antiparkinsonism drugs
T43*	Poisoning by psychotropic drugs, not elsewhere classified
T44*	Poisoning by drugs primarily affecting the autonomic nervous system
T45*	Poisoning by primarily ststemic and haematological agents, not elsewhere classified
T46*	Poisoning by agents primarily affecting the cardiovascular system
T47*	Poisoning by agents primarily affecting the gastrointestinal system
T48*	Poisoning by agents primarily acting on smooth and skeletal muscles and the respiratory system
T49*	Poisoning by topical agents primarily affecting skin and mucous membrane and by ophthalmological, otorhinolaryngological and dental drugs
T50*	Poisoning by diuretics and other unspecified drugs, medicaments and biological substances
T96*	Sequelae of poisoning by drugs, medicaments and biological substances
X40*	Accidental poisoning by and exposure to nonopioid analgesics, antipyretics, and antirheumatics including acetominophen
X41*	Accidental poisoning by and exposure to antiepileptic, sedative hypnotic, antiparkinsonism, psychotropic drugs NEC
X42*	Accidental poisoning by and exposure to narcotics and psychodysleptics (hallucinogens) NEC (includes diamorphine (heroin), cocaine, codeine, lysergide (LSD), morphine, pethidine, narcotic NEC, hallucinogen NEC, hydrocodone, oxycodone (oxycontin) and fentanyl, among others)
X43*	Accidental poisoning by and exposure to other drugs acting on the autonomic nervous system
X44*	Accidental poisoning by and exposure to other and unspecified drugs, medicaments, and biological substances
Y10*	Poisoning by and exposure to nonopioid analgesics, antipyretics and antirheumatics, undetermined intent

Vaccine-associated

A80.0	Acute paralytic poliomyelitis, vaccine-associated
M02.2	Postimmunization arthropathy
T88.0	Infection following immunization
T88.1	Infection complications following immunization
Y58*	Drugs, medicaments and biological substances causing adverse effects in therapeutic use - Bacterial vaccines

ADE very likely

A04.7	Enterocolitis due to Clostridium difficile
E15	Nondiabetic hypoglycaemic coma
H91.0	Ototoxic hearing loss
K52.1	Toxic gastroenteritis and colitis
K71*	Toxic liver disease

L51*	Erythema multiforme
L56.2	Photocontact dermatitis [berloque dermatitis]
N99.0	Postprocedural renal failure
O74.2	Cardiac complications of anaesthesia during labour and delivery
O74.3	Central nervous system complications of anaesthesia during labour and delivery
Y63*	Failure in dosage during surgical and medical care
Y65.1	Wrong fluid in infusion
Y69	Unspecified misadventure during surgical and medical care

ADE, adverse drug event; NEC, not elsewhere classified
(*) denote including sub-codes.
